# EGFL6 promotes colorectal cancer cell growth and mobility and the anti‐cancer property of anti-EGFL6 antibody

**DOI:** 10.1186/s13578-021-00561-0

**Published:** 2021-03-16

**Authors:** Ting-Yi Sung, Han-Li Huang, Chun-Chun Cheng, Fu-Ling Chang, Po-Li Wei, Ya-Wen Cheng, Cheng-Chiao Huang, Yu-Ching Lee, Wei-Chun HuangFu, Shiow-Lin Pan

**Affiliations:** 1 Ph.D. Program in Drug Discovery and Development Industry, College of Pharmacy, Taipei Medical University, 11031 Taipei, Taiwan; 2 TMU Biomedical Commercialization Center, Taipei Medical University, 11031 Taipei, Taiwan; 3 TMU Research Center of Cancer Translational Medicine, Taipei Medical University, No. 250, Wuxing St., 11031 Taipei, Taiwan; 4 Graduate Institute of Cancer Biology and Drug Discovery, College of Medical Science and Technology, Taipei Medical University, No. 250, Wuxing St., 11031 Taipei, Taiwan; 5 Division of Colorectal Surgery, Department of Surgery, Taipei Medical University Hospital, Taipei Medical University, 11031 Taipei, Taiwan; 6 Translational Laboratory, Department of Medical Research, Taipei Medical University Hospital, 11031 Taipei, Taiwan; 7 Department of Surgery, College of Medicine, Taipei Medical University, Taipei, Taiwan; 8 Ph.D. Program for Cancer Molecular Biology and Drug Discovery, Taipei Medical University, Taipei, Taiwan; 9 Division of Breast Surgery, Department of Surgery, Taipei Medical University Hospital, No. 252, Wuxing St., 11031 Taipei, Taiwan

**Keywords:** EGFL6, Colorectal cancer, EGFR/αvβ3, Tumor progression, Therapeutic antibody

## Abstract

**Background:**

The availability of a reliable tumor target for advanced colorectal cancer (CRC) therapeutic approaches is critical since current treatments are limited. Epidermal growth factor-like domain 6 (EGFL6) has been reported to be associated with cancer development. Here, we focused on the role of EGFL6 in CRC progression and its clinical relevance. In addition, an anti-EGFL6 antibody was generated by phage display technology to investigate its potential therapeutic efficacy in CRC.

**Results:**

EGFL6 expression significantly increased in the colon tissues from CRC patients and mice showing spontaneous tumorigenesis, but not in normal tissue. Under hypoxic condition, EGFL6 expression was enhanced at both protein and transcript levels. Moreover, EGFL6 could promote cancer cell migration invasion, and proliferation of CRC cells via up-regulation of the ERK/ AKT pathway. EGFL6 also regulated cell migration, invasion, proliferation, and self-renewal through EGFR/αvβ3 integrin receptors. Treatment with the anti-EGFL6 antibody EGFL6-E5-IgG showed tumor-inhibition and anti-metastasis abilities in the xenograft and syngeneic mouse models, respectively. Moreover, EGFL6-E5-IgG treatment had no adverse effect on angiogenesis and wound healing

**Conclusions:**

We demonstrated that EGFL6 plays a role in CRC tumorigenesis and tumor progression, indicating that EGFL6 is a potential therapeutic target worth further investigation.

**Supplementary Information:**

The online version contains supplementary material available at 10.1186/s13578-021-00561-0.

## Introduction

Colorectal cancer (CRC) is the third most commonly diagnosed malignant cancer with high morbidity and mortality in the world. Approximately 50% CRC patients will develop to metastatic CRC, and about 25% patients have become metastasis already upon diagnosed [1]. Bevacizumab (Avastin®, Genentech) is a monoclonal antibody targeting vascular endothelial growth factor (VEGF), it generally combine with 5-fluorouracil (5-FU)-based chemotherapy regimens as the first and second-line treatment for metastatic CRC (mCRC). Despite significant improvement in progression-free survival (PFS) and overall survival (OS) showed in mCRC patients receiving bevacizumab plus chemotherapy compared to chemotherapy alone [1, 2], disease progression still arises within 9 months in patients [3]. The incidence of side effects, such as perforation, hemorrhage, and wound healing complications associated with VEGF inhibition, also increased when use bevacizumab in elderly and female patients [4, 5]. CRC patients who received stent placement to treat malignant bowel obstruction may also have a higher risk of colon perforation [6]. Therefore, an effective therapeutic agent with tolerable adverse effect is an urgent medical need for CRC.

Cancer cells tend to release factors to conserve a microenvironment that is favorable for them to growth [7]. It has been demonstrated that some proteins such as Ca^2+^ binding EGF (CB-EGF)—like repeat-containing proteins, were expressed in high level during development and then were often reactivated under pathological conditions like cancer [8]. Epidermal growth factor-like domain 6 (EGFL6) is a member of the epidermal growth factor (EGF) repeats protein superfamily, EGFL6 is suggested as a secreted form protein due to its putative signal peptide and implied the potential binding activities with integrins via its arginine-glycine-aspartic acid (RGD) motif [9]. Previous studies have shown that EGFL6 is highly expressed in fetal tissues, but the expression is dramatically reduced in adult tissues [9, 10]. The role of EGFL6 was previously shown to promote endothelial cell migration, angiogenesis during bone development [11], and promote adipose tissue-derived stromal vascular cell proliferation [12].

Recent evidence from human tumor biopsy transcription analysis has shown that EGFL6 mRNA is expressed at high levels in various cancers, including CRC, while levels in normal tissues were nearly undetectable [9, 13, 14, 15]. However, the role of EGFL6 in promoting CRC tumorigenesis and progression still needs to be determined. In this study, we focus on investigating the clinicopathologic role of EGFL6 in CRC patients and its involvement in tumorigenesis, as well as its function in regulating cell proliferation, migration and underling signaling pathways in CRC. Moreover, we developed an anti-EGFL6 antibody, EGFL6-E5-IgG, to verify the anti-tumor, anti-metastatic and anti-angiogenic properties *in vivo* to validate the potential of EGFL6 as a therapeutic target in CRC.

## Results

### EGFL6 increased abundance in CRC

**Fig. 1:**
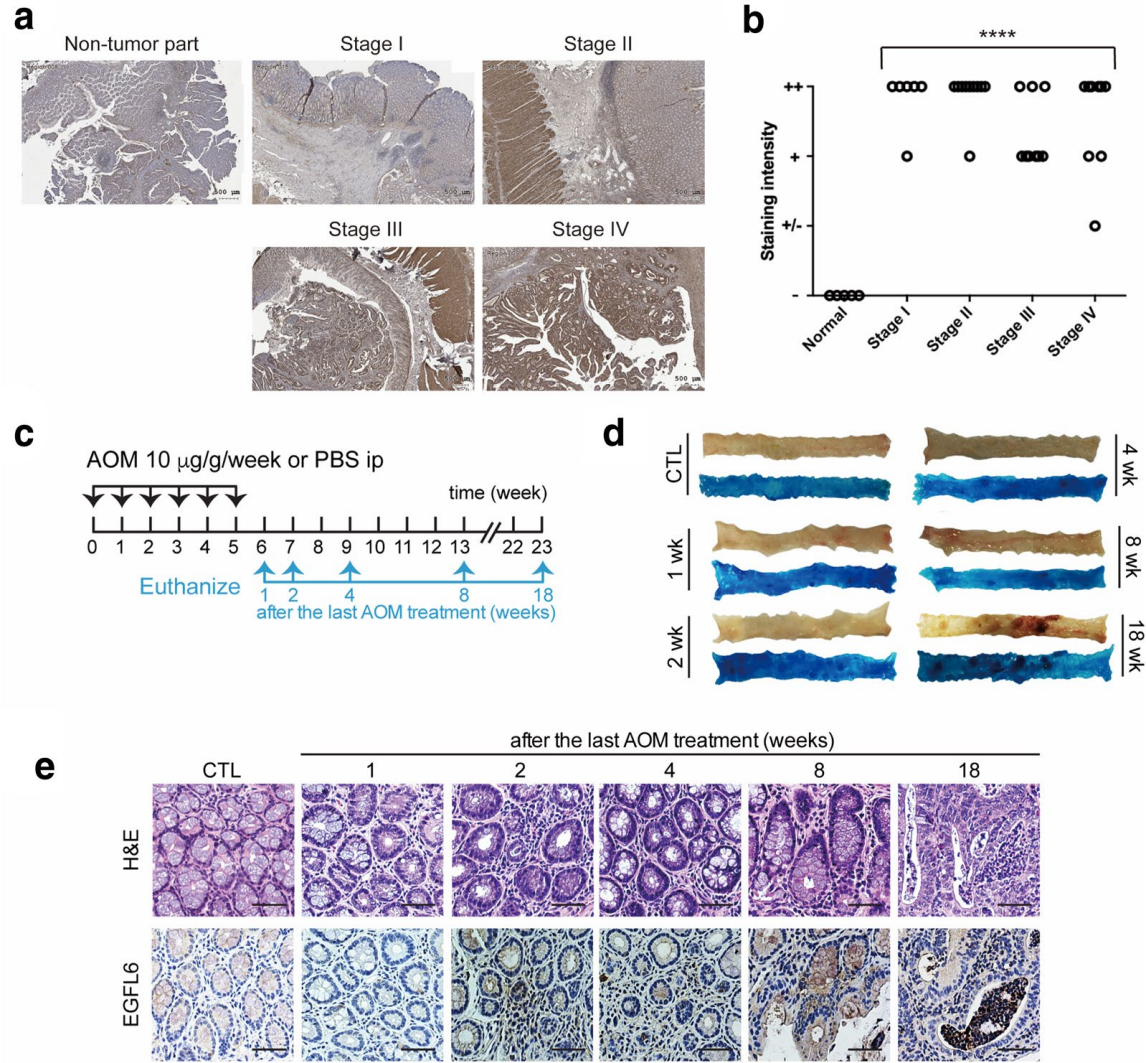
EGFL6 expresses in CRC patients and in early-developed colon carcinogenesis animal model. **a** EGFL6 (Epidermal growth factor-like protein 6) expression in human CRC (colorectal cancer) tissue with indicated stages under 100X magnification. EGFL6 staining score was “++” for stage I to IV. **b** EGFL6 staining scoring of non-tumor part and tumor part were showed. Normal group (n = 5) and human CRC group stage I (n = 6), stage II (n = 10), stage III (n = 9), stage IV (n = 9). **c** AOM (Azoxymethane) mouse model schedule. Total number of 35 eight-week-old A/J mice were randomly divided into control or AOM treatment group. Mice were sacrificed and colons were collected at 1, 2, 4, 8, and 18 weeks after the last AOM challenge to assess for methylene blue, H&E (hematoxylin and eosin stain) staining, and IHC (immunohistochemistry) staining of EGFL6. **d** Methylene blue staining of intestinal tissue (from anus to the cecum) to observe changes in the intestines. Stained with 0.05% methylene blue. **e** IHC staining of intestinal tissues in different time points after AOM stimulation. Scale bar represents 50 µm. **** *p* < 0.0001

Many oncogenic proteins were highly expressed during development and then were reactivated in adult under pathological conditions. The aberrant expression of EGFL6 in adult tissue might be related to tumorigenesis. To prove our hypothesis, we analyzed the EGFL6 expression level in patients’ tumor tissues by using IHC staining (Fig. 1a, b). To score EGFL6 expression, staining intensity was evaluated and scored by pathologists in Changhua Christian Hospital in Taiwan. The scoring rate “−” indicated negative, “−/+” and “+” were classified as low-expression, “++” was classified as high expression. The result showed that EGFL6 expression could be detected in each CRC stage, but not in normal tissue in CRC patients (Fig. 1a, b). After compared with the healthy colon tissue, the statistical analysis result demonstrated that EGFL6 expression correlated with CRC (*p* < 0.0001) (Fig. 1b). Besides, EGFL6 expression was not associated with tumor size, nearby lymph nodes involved, or tumor metastasis (Table 1). We conducted an experimental azoxymethane (AOM)-induced mouse model to validate the role of EGFL6 in tumor progression *in vivo* (Fig. 1c). AOM-induced mice showed aberrant crypt foci at 8 and 18 weeks after the last AOM injection. These crypt foci are putative precursor lesions for CRC according to methylene blue staining (Fig. 1d). We further demonstrated that the expression of EGFL6 was notably accompanied with CRC progression during spontaneous tumorigenesis (Fig. 1e).

**Table 1:** Association of EGFL6 expression and clinical parameters in tumor tissues of CRC patients

Parameters	EGFL6		
	Low	High	p value
	(n = 73)	(n = 119)	
Age (years)			
≦ 65	32	62	
> 65	41	57	0.299
Gender			
Female	30	60	
Male	43	59	0.235
T factor			
1	3	4	
2	10	18	
3	43	67	
4	17	30	0.968
N factor			
0	31	52	
1 + 2	42	67	0.882
M factor			
0	58	101	
1	15	18	0.333
Stage			
I	10	13	
II	18	35	
III	29	53	
IV	16	18	0.555

*T factor* tumor size,* N factor* lymph nodes,* M factor* metastasis

### EGFL6 plays a role in promoting cell proliferation

**Fig. 2:**
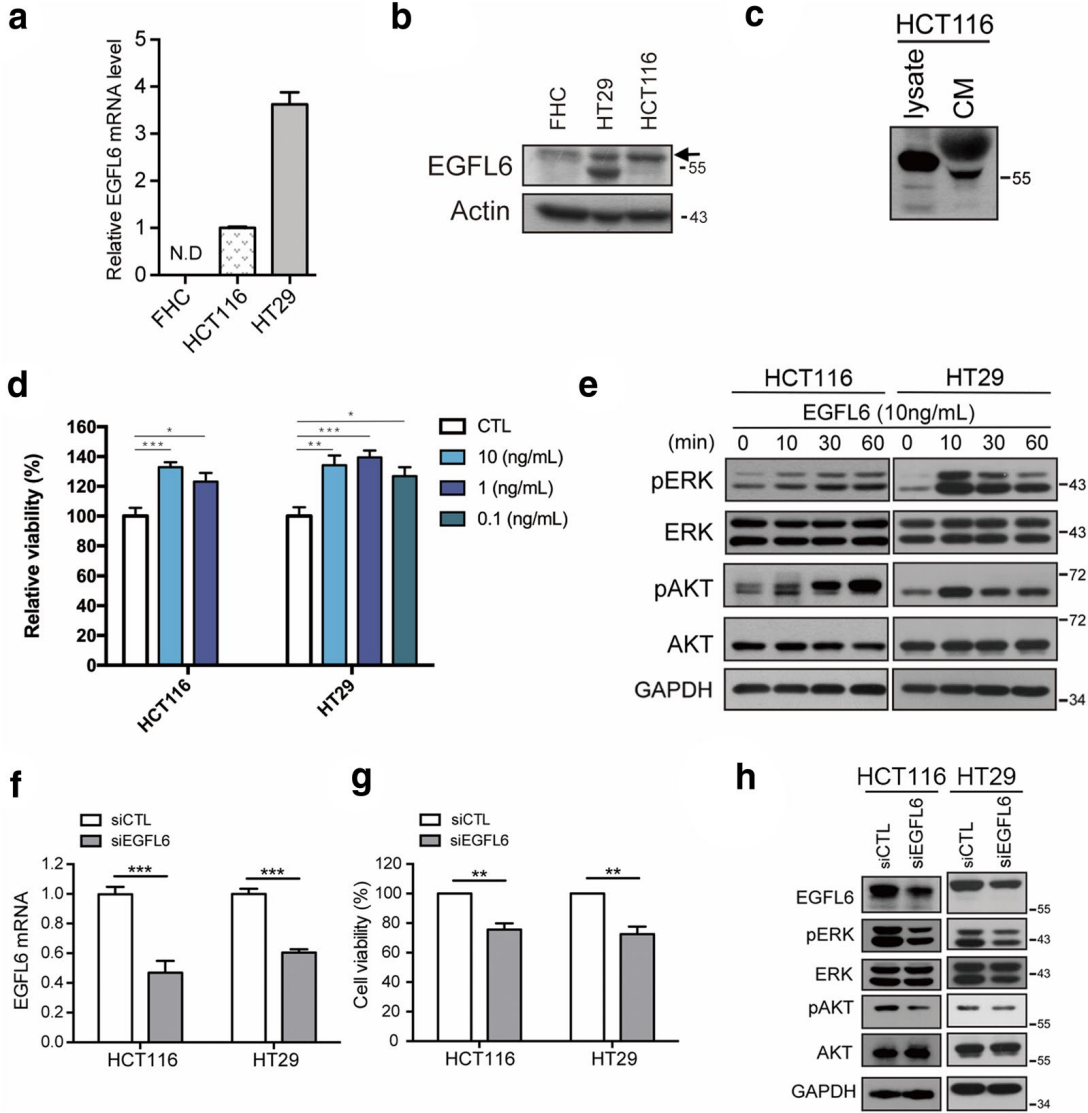
EGFL6 promotes CRC cell proliferation. **a**, **b** Relative EGFL6 mRNA level and protein expression of FHC (human normal colon epithelial cell), HCT116 (human colorectal carcinoma epithelial cell) and HT29 (human colorectal epithelial adenocarcinoma cell). **c** EGFL6 expression of HCT116 culture medium presenting in western blot. CM: condition medium. **d** The relative viability of HCT116 (2 $\times$ 10^3^ cell/well) and HT29 (3 $\times$ 10^3^ cell/well) treated from various concentrations of human recombinant EGFL6 (0.1, 1 or 10 ng/mL), incubated 5 days in 96 well for SRB. **e** Cell proliferation signals (p-ERK (extracellular signal–regulated kinase), p-AKT (protein kinase) protein expression after EGFL6 (10 ng/mL) treatment by different time point. **f** *EGFL6* mRNA expression of HCT-116 and HT29 after knockdown *EGFL6*. Incubated 48 h after siRNA treatment. **g**, **h** Tumor cell viability as well as ERK and AKT phosphorylation of HCT-116 and HT29 after *EGFL6* knockdown. Incubated 48 h after siRNA treatment. *p < 0.05, **p < 0.01, ***p < 0.005

To investigate the role of EGFL6 in CRC *in vitro*, we first examined the EGFL6 expression level in different CRC cell lines compare to the normal colon epithelial cell. We found that in protein level, EGFL6 was highly expressed in CRC cells except in SW480, and EGFL6 expressed in very low level in normal colon epithelial cells. Since HCT116 and HT29 express EGFL6 more abundantly compare to other CRC cell lines, we use HCT116 and HT29 for further investigation (Fig. 2a, b, Additional file 1: Figure S1). EGFL6 was also detected in the culture medium, implying EGFL6 is a secreting protein (Fig. 2c). The migration of EGFL6 was slightly slower in culture medium due to N-linked glycosylation modification of EGFL6 [9]. A specific glycosylation site of EGFL6 is at Asn397 as described in GeneCards. To investigate the role of EGFL6 in tumorigenesis, we examined if EGFL6 could induce cell proliferation. EGFL6 induced CRC cell proliferation when treated with human recombinant EGFL6 protein (Fig. 2d). In addition, EGFL6 activated extracellular signal–regulated kinase (ERK) and protein kinase B (AKT) phosphorylation, which mediated cell proliferation and is associated with CRC progression (Fig. 2e, Additional file 1: Figure S2). We then evaluated the impact of *EGFL6* knockdown on the growth of CRC cells. *EGFL6* siRNA #10 showed a significant silencing effect with about 50% *EGFL6* mRNA knockdown in CRC cells in comparison of scrambled siRNA (Fig. 2f, g). *EGFL6* knockdown using siEGFL6 was associated with reduced tumor cell viability as well as ERK and AKT phosphorylation (Fig. 2h, Additional file 1: Figure S2, S3). These data purported that EGFL6 potentially promotes cancer cell proliferation through ERK and AKT pathways.

**Fig. 3:**
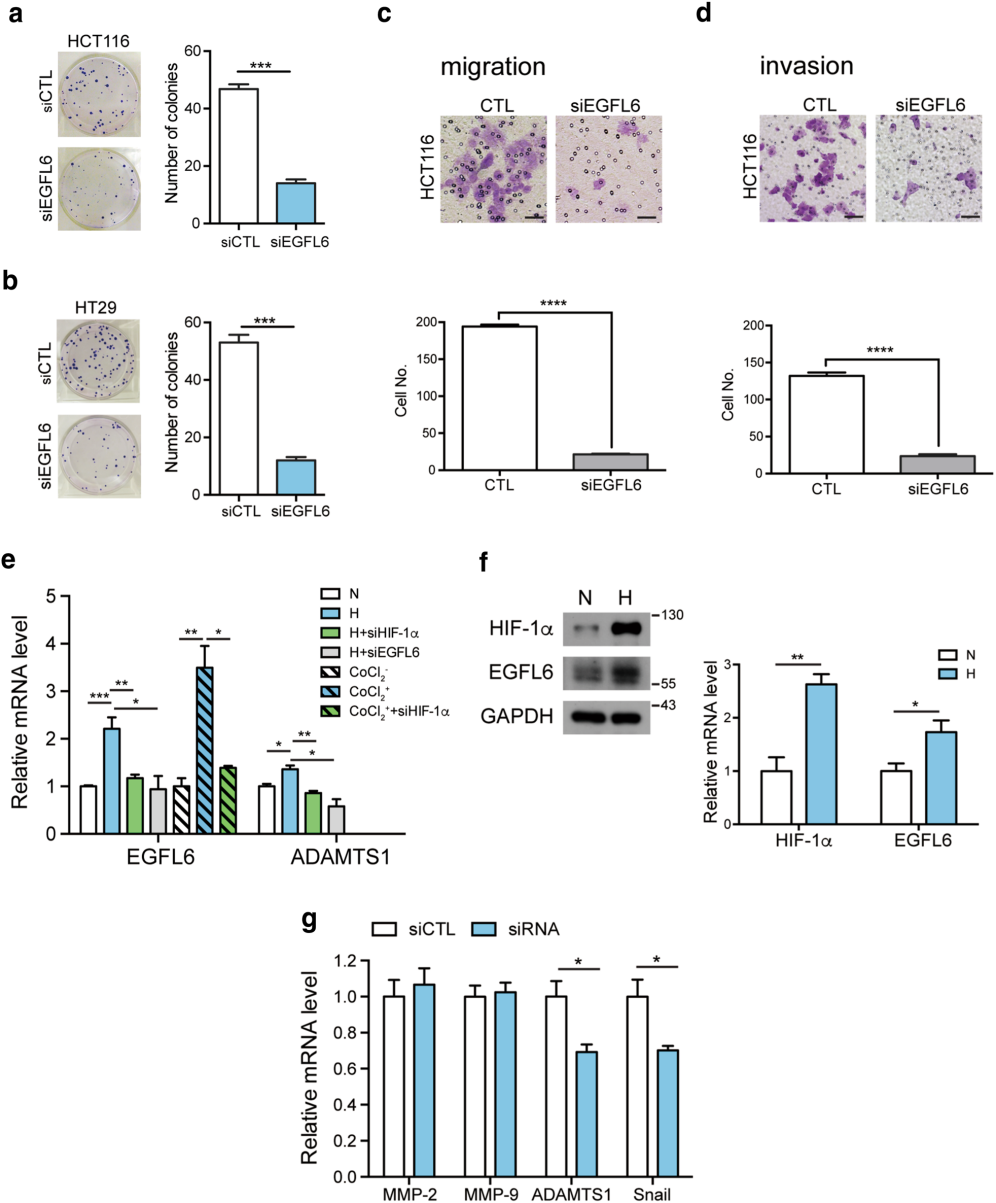
EGFL6 regulates CRC cell migration and invasion. **a**, **b** Colony formation assay to test the proliferation of HCT116 and HT29 after silencing *EGFL6*. **c**, **d** The migration and invasion assay of HCT116 cell after silencing *EGFL6*. Scale bar represents 100 µm. **e** The mRNA level of *EGFL6* and *ADAMTS1 (a disintegrin and metalloprotease with thrombospondin motif 1*) under normoxia and hypoxic conditions to validate the signaling relationship between *EGFL6* and *HIF-1α*. N = normoia, H = hypoxia. **f** HIF-1α and EGFL6 expression under hypoxia condition. N = normoia, H = hypoxia. **g** The mRNA expression of invasion and migration-associated *MMP (*matrix metalloproteinase*)-2, MMP-9, ADAMTS1* and *Snail* after silencing EGFL6. *p < 0.05, **p < 0.01, ***p < 0.005, ****p < 0.0001

### EGFL6 plays roles in cancer cells migration and invasion and is inducible under hypoxia environment

Next, we test whether silencing *EGFL6* in CRC cells affects the clonogenic potential. The data showed that CRC cells treated with *EGFL6* siRNA exhibited fewer colony numbers compared with cells treated with the scramble siRNA (Fig. 3a, b). Clonogenic potential relates to the cell proliferation ability, whether migration and invasion are also crucial abilities for cancer cell to develop. Since cell migration and invasion are critical properties for the dissemination of cancer cells and metastasis; therefore, *in vitro* migration and invasion assays were performed to investigate the effect of EGFL6 on cell invasiveness. There were significantly fewer migrated cells in *EGFL6* siRNA transfected groups compared with scrambled siRNA-treated groups (Fig. 3c). Consistent with the finding in migration assay, *EGFL6* siRNA treated cancer cells reaching the lower matrigel-free well was significantly reduced in comparison with scrambled siRNA-treated groups (Fig. 3d).

Hypoxia is a key regulatory factor in tumor growth, survival and proliferation when cancer cells experience genetic changes in the toxically hypoxic environment. We examined whether EGFL6 could be induced under hypoxia condition in HCT116 and both transcript and protein levels of EGFL6 were enhanced by hypoxia condition (Fig. 3e, f). Moreover, hypoxia-induced *EGFL6* expression was abrogated under *hypoxia-inducible factor 1α* (*HIF-1α*) siRNA treatment. To corroborate this result, CRC cells were treated with CoCl_2_, a hypoxia mimicking agent reported to stabilize HIF-1α. CoCl_2_ treatment enhanced *EGFL6* expression, whereas *EGFL6* expression was reduced when given the *HIF-1α* siRNA treatment in CoCl_2_ condition (Fig. 3e). Furthermore, *a disintegrin and metalloproteinase with thrombospondin motif (ADAMTS)* is an acute hypoxia-inducible gene, the data showed that *ADAMTS1*’s expression was enhanced under hypoxia and reduced with *HIF-1α* siRNA treatment. *ADAMTS1* expression was also inhibited with *EGFL6* siRNA treatment under hypoxia (Fig. 3e). Therefore, EGFL6, as well as its downstream pathway, such as ADAMTS1, can respond to hypoxia condition. This also suggests the potential role of EGFL6 in regulating cancer cell migration under hypoxia condition.

In addition, matrix metalloproteinase (MMP), ADAMTS1 and Snail are factors associated with cancer migration and invasion; thus, we analyzed *MMP-2*, *MMP-9*, *ADAMTS1*, and *Snail* expressions in *EGFL6* siRNA treated HCT116 and HT29 (Additional file 1: Figure S4). It showed that EGFL6 knockdown did not affect the gene expression of *MMP* while *ADAMTS1* were both significantly reduced in HCT116 and HT29, another related factor—*Snail* was significantly reduced in HCT116 *EGFL6* siRNA treated cells at mRNA level (Fig. 3g). Taken together, these results have proven that EGFL6 plays roles in invasive and migration for CRC cells.

### EGFL6 activates cancer cell signaling through EGFR and integrin receptors

**Fig. 4:**
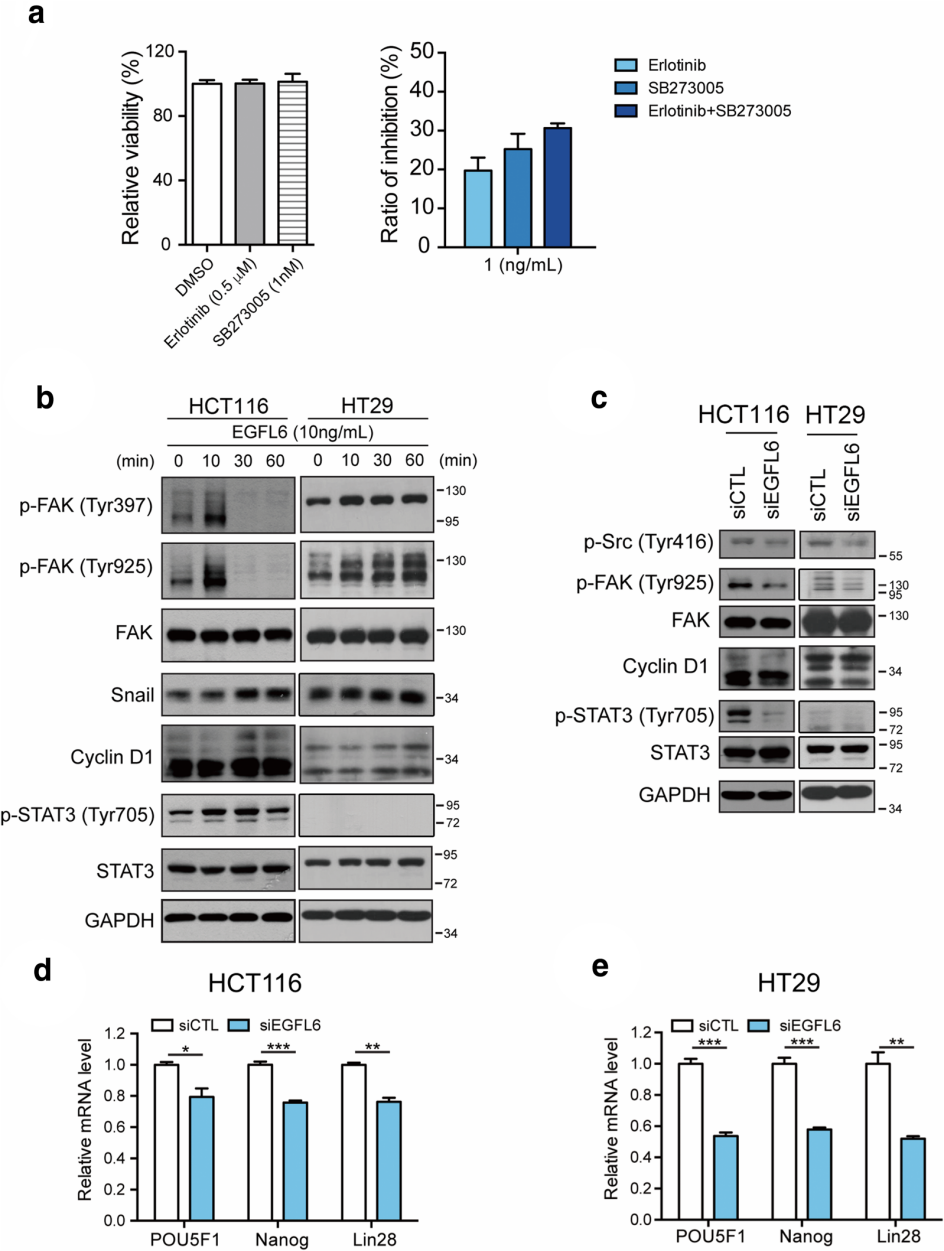
EGFL6 activates EGFR and integrin signaling in CRC cells. **a** EGFL6-induced cell proliferation ability under SB273005 (an αvβ3 integrin inhibitor), and Erlotinib (a receptor tyrosine kinase inhibitor on EGFR (epidermal growth factor receptor) treatment in HCT116 cell line. **b**, **c** The migration, invasion and proliferation-associated protein signaling of HCT116 and HT29 after EGFL6 treatment by time and after silencing of *EGFL6*. **d**, **e** The mRNA expression of cell stemness maintenance associated gene, *POU5F1 (POU class 5 homeobox 1)*, *NANOG* and *LIN28* after EGFL6 silencing in HCT116 and HT29. *p < 0.05, **p < 0.01, ***p < 0.005

Since EGFL6 harbors EGF repeats domain and RGD motif, EGFL6 might induce cell proliferation through its EGF repeats coupling with epidermal growth factor receptor (EGFR) or through its RGD motif binding to integrin receptors. We demonstrated that EGFL6-induced cell proliferation was inhibited when cells were treated with Erlotinib, a receptor tyrosine kinases (RTKs) inhibitor which acts on EGFR. It has been reported that many cancer cells express αvβ3 integrins. Similar results were shown that EGFL6-induced cell proliferation was reduced in the presence of αvβ3 integrin inhibitor SB273005 (Fig. 4a). The inhibitory ability was clear when combining Erlotinib and SB273005 (Fig. 4a). Hence, we supposed that EGFL6 affects cell proliferation capacity through EGFR/integrin receptors.

Transmembrane growth factor receptors, such as EGFR, and integrin receptors form multi-protein signaling complexes with focal adhesion kinase (FAK) [16]. FAK auto-phosphorylation at Tyr397 creates a binding site for the Src-homology 2 (SH2) domain, which promotes Src family kinases phosphorylate FAK at Tyr925 [17, 18]. We found that EGFL6 was associated with FAK-Src signaling in which phosphorylated FAKs (Tyr397 and Tyr925) and Src phosphorylation at Try416 was reduced after treating siEGFL6 (Fig. 4b, c). In addition, AKT, FAK and Src kinase collaboration was involved in CRC cell migration and invasion [19]. Here, we showed that phosphorylation of ERK, AKT and Snail expression were associated with EGFL6 induction (Figs. 2e and 4b). FAK plays a role in the regulation of cell cycle progression, correlating with changes in cyclin D1 expression, and cyclin D1 deregulation may promote tumor development [20, 21]. EGFL6 can affect cyclin D1 in which cyclin D1 was enhanced with EGFL6 treatment, but decreased in the presence of EGFL6 siRNA (Fig. 4b, c). Further, signal transducer and activator of transcription 3 (STAT3), FAK and Src are known to regulate cancer stem cell proliferation and self-renewal [22, 23]. It is shown that STAT3 phosphorylation was enhanced after EGFL6 treatment, but was reduced after *EGFL6* siRNA treatment (Fig. 4b, c). From this result, we believe EGFL6 expression may associate with cancer cell self-renewal, and colony forming ability is related to the cell self-renewal ability [24] (Fig. 3a, b), we further explored the mechanism of EGFL6 in keeping the cell stemness by detecting the expression of several associated genes, including *POU class 5 homeobox 1 (POU5F1)*, *NANOG*, and *LIN28*. The mRNA level of *POU5F1*, *NANOG*, and *LIN28* all had obvious reductions when *EGFL6* was knocked down in both HCT116 and HT29 (Fig. 4d, e). Taken together, we found that EGFL6 could regulate cancer cell migration, invasion, proliferation and self-renewal by affecting EGFR and integrin receptor signaling. The quantification of the western blot signals above were showed in Additional file 1: Figure S2.

### EGFL6 antibody demonstrated anti‐cancer and anti‐metastasis properties without interfering wound healing in vivo

According to the above data, we assume that EGFL6 might be a potential therapeutic target for CRC treatment. In order to address the *in vivo* function of EGFL6, EGFL6-E5-IgG, a humanized antibodies specific targeting to EGFL6 were generated using a phage display system.

**Fig. 5:**
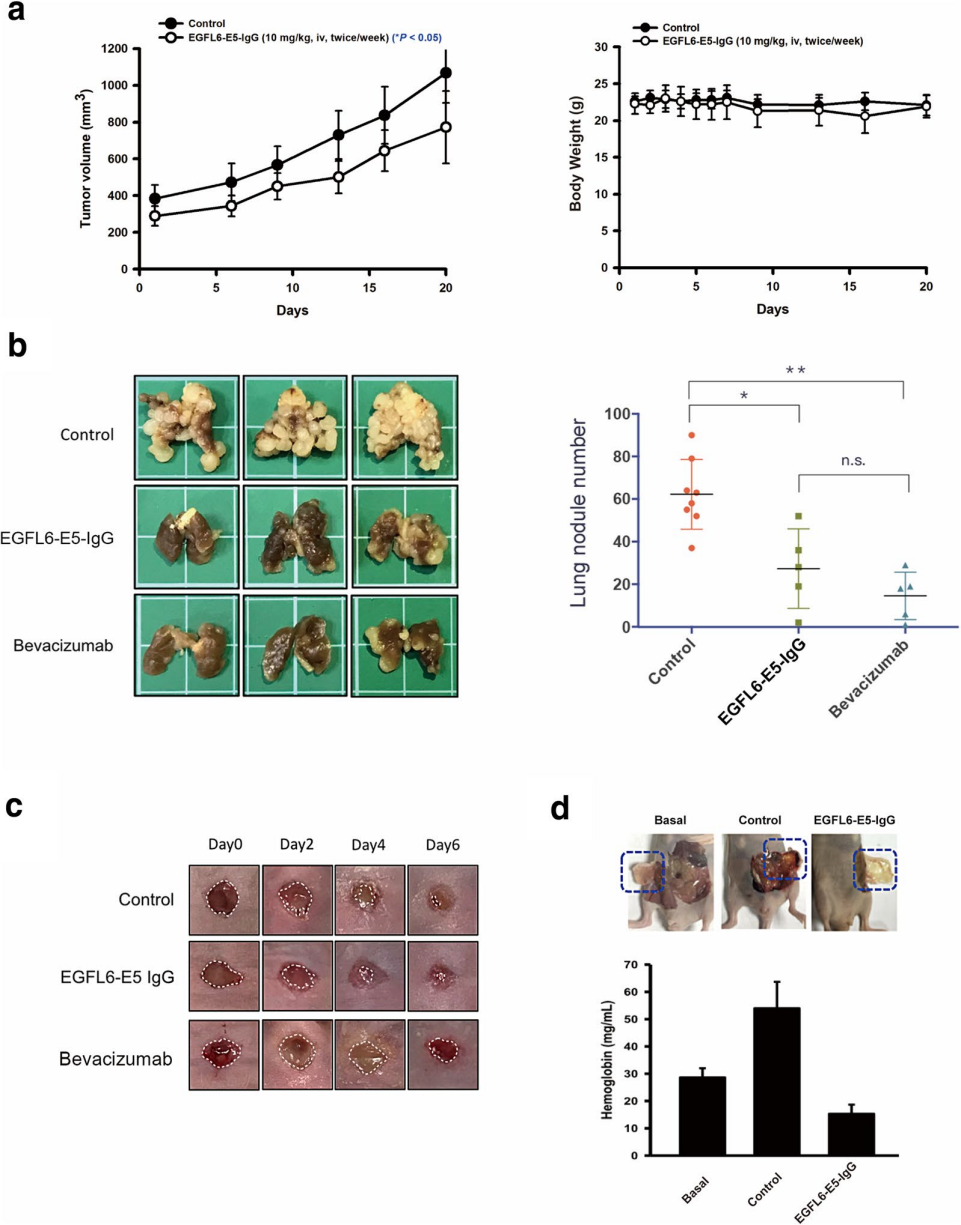
*In vivo* efficacy evaluation of EGFL6 antibody. **a** 16 six-week-old nude mice were injected subcutaneously with the same volume of Matrigel, and 1$\times$10^7^ of HCT-116 cells into the right flank of each animal. The tumor volume and body weight observation in HCT-116 xenograft model treated with two groups: control (IgG, i.p, twice/week, n = 8) and EGFL6-E5-IgG (10 mg/kg, iv, twice/week, n = 8). **b** Eight-week-old balb/c mice (NLAC, Taipei, Taiwan) were injected with CT-26 cells (2$\times$10^5^ cell/mouse) intravenously and then divided into three groups for treatments: control (IgG, 20 mg/kg, n = 8), EGFL6-E5-IgG (20 mg/kg, n = 6) and bevacizumab (20 mg/kg, n = 6). Antibodies were given every 5 days by tail vein injection. Body weight was monitored every 2 or 3 days. Scale bar represents 1 cm. **c** A total of 11 seven-week-old nude mice (NLAC, Taipei, Taiwan) were used in wound healing model, 3 groups of mice treated with Control IgG (20 mg/kg, n = 5), EGFL6-E5-IgG (20 mg/kg, n = 5), and bevacizumab (20 mg/kg, n = 5). Treatments were given intravenously for a consecutive five-on-two-off regimen. Observed for 1 week until Control healed. **d** A total of twelve nude mice (NLAC, Taipei, Taiwan) were divided into four groups. For basal group (n = 2), mice were injected with 500 µl matrigel subcutaneously. For control group (n = 5), mice were injected with 500 µl matrigel subcutaneously mixed with endothelial growth factor (EGF, 150 ng/mL) and heparin (10 µl). For indicated antibody group (n = 5), mice were injected with 500 µl matrigel subcutaneously mixed with endothelial growth factor (EGF, 150 ng/mL), heparin (10 µl), and treated anti-EGFL6 antibody (15 mg/kg, iv, q5D). *p < 0.05, **p < 0.01

To verify the anti-cancer activity of EGFL6-E5-IgG, HCT-116 xenograft model was established. EGFL6-E5-IgG inhibited tumor growth in HCT-116 xenograft model with TGI 36.2% (**p < 0.01), without significant body weight change (Fig. 5a), indicate EGFL6-E5-IgG’s anti-tumor capability in CRC. Along with the association (k_on_) and dissociation (k_off_) rates, the affinity (K_D_) of EGFL6-E5-IgG was calculated to be 1.91 $\times$ 10^−8^ M (Table 2 and Additional file 1: Figure S5). The *in vitro* data using HCT116 treated by EGFL6-E5-IgG for cell proliferation and migration assay were presented in Additional file 1: Figure S6. The anti-cancer activity of EGFL6-E5-IgG was also conducted in another xenograft model (human glioblastoma), the data can be found in Additional file 1: Figure S7.

**Table 2:** k_on_ and k_off_ rate constants of E5 IgG targeting to EGFL6

Ligand	Analyte (IgG)	k_on_(10^3^ M^− 1^S^− 1^)	k_off_(10^− 5^S^− 1^)	K_D_(10^− 8^ M)	χ^2^
EGFL6	E5	2.3 ± 0.00251	4.38 ± 0.00177	1.91 ± 0.0792	67.39

k_on_, association rate; k_off_, dissociation rate; KD, affinity binding constants; χ2, Chi-squared test

EGFL6-E5-IgG was then used to evaluate its anti-metastatic property in a syngeneic lung metastasis model. Both EGFL6-E5-IgG and bevacizumab significantly inhibited CT-26 metastasis compared to the Control group (* *p* < 0.05 and ** *p* < 0.01, respectively) without significant differences between two treated groups (Fig. 5b), indicating that EGFL6 blockade can suppress CRC cell metastasis *in vivo* and EGFL6-E5-IgG’s potential effectiveness.

Protracted wound healing is one of the major side effects interfering the treatment efficacy of bevacizumab. EGFL6-E5-IgG-treated animals have similar wound closure speed compared to the Control group. On day 6, wounds of Control and EGFL6-E5-IgG groups already formed scabs, but not in bevacizumab-treated group (Fig. 5c), suggesting that bevacizumab has the potential to affect wound repairing while EGFL6-E5-IgG exhibited no impact on wound healing. Here we use angiogenesis assay to demonstrate EGFL6-E5-IgG has potential to inhibit angiogenesis, despite no statistically significant showed, hemoglobin concentration still lower than the Control group (Fig. 5d). These data indicated the anti-cancer, anti-metastatic, anti-angiogenesis capability as well as no impact on wound healing, suggested that EGFL6-E5-IgG has the potential to become a therapeutic agent for CRC.

## Discussion

The current CRC first-line treatment is bevacizumab combined with chemotherapy, such as 5-FU, oxaliplatin or irinotecan. The regimens evidently improve the overall and progression-free survival for CRC patients. However, it’s worth to note that in a meta-analysis review article, there are some statistically significant increase in severe adverse events associated with bevacizumab, for example, severe hypertension and gastrointestinal perforation [25]. In addition, Koichi Taira et al. reported that patient received bevacizumab occurred skin ulcer and wound healing delayed, and suggest the treatment of bevacizumab should be suspended at least 5 weeks before a patient undergo an operation [26]. Furthermore, drug resistance also occurs using bevacizumab [27], leading to treatment failure. Although these adverse events don’t happen often, but they are potentially life threatening, which need to be considered in any decision. Therefore, finding new targets and developing therapeutic strategy towards CRC is still an important issue.

EGFL6 is proved to relate to the progression of several cancers [28–31]; however, the function and mechanism of EGFL6 in CRC has not yet been elucidated in detail. Recently, a research group reportedEGFL6 gene was upregulated in human CRC tissues by gene analysis, but were nearly undetected in normal colorectal tissues [32]. EGFL6 also significantly express high amount in oral squamous cell carcinoma tumor part, but not express in normal part, with significantly higher expression in stage IV compare to stage I [28]. The data in this research exhibited statistically significant high EGFL6 expression in stage I-IV patient tumor tissues, without expression in non-tumor tissue, in accordance with previous researches. Notably, in the AOM-induced mice model, EGFL6 started to express when colorectal polyps occurred, and the expression of EGFL6 increased as colorectal polyps progressed. Hence, we speculated that EGFL6 expression could be detected in the precancerous stage of CRC. As colorectal polyps usually progress to colorectal tumors [33], EGFL6 could be as a suitable biomarker for early CRC detection. According to these results, we assumed that EGFL6 presumably functioned as a tumor-specific protein and could promote tumorigenesis.

Recently, some studies have showed EGFL6 could modulate cancer cell proliferation, and metastasis in colorectal cancer and different cancer types [13, 15, 29, 31]. From the data in this research, knockdown of EGFL6 is associated with tumor cell viability reduction as well as reduced ERK and AKT phosphorylation. That indicates EGFL6 promotes CRC cells survival and proliferation through ERK and AKT pathways, which was consistent with previous report [13]. The same result was also found in zebrafish model [34], but was different from Zhang‘s study, in which EGFL6 recombinant protein did not change p-ERK or p-AKT level in CRC cell HCT116 and SW480 [15]. The possible explanation of this difference might due to different treatment procedures. In Zhang‘s study, CRC cells were treated with 500 ng/mL EGFL6 for 48 h, whereas we treated only 10 ng/mL for 60 min and the ERK and AKT phosphorylation were obviously activated. In Zhang’s study, they found that EGFL6 modulates cell proliferation through WNT/β-catenin pathway. Here, we discovered that p-AKT was increased by EGFL6, which means EGFL6 may contribute to cell proliferation pathway. Whereas, EGFL6 was found to contribute to CRC cell proliferation in Zhang’s study, which is consistent with the result from this research.

To further understand the underlining mechanism of EGFL6 mechanism in CRC, we conducted a series of experiments to investigate the EGFL6 downstream pathways. The *in vitro* studies proffered evidence that under hypoxia condition, EGFL6 binds to EGFR, activates EGFR/αvβ3 integrin receptors to affect cancer cell proliferation. Furthermore, Src, FAK and STAT3, which kinases are related to cell proliferation, migration and invasion, were phosphorylated by EGFL6 in CRC cell. This result coincides with the research that the collaboration of AKT, FAK and Src was associated with migration and invasion of CRC cells [19]. Cyclin D1, activated by ERK, was also found associated with EGFL6 expression. We demonstrated in detail that EGFL6 modulates CRC cell proliferation, self-renewal ability, cell migration and invasion via EGFR/αvβ3 integrin signaling.

Hypoxia is a condition that could be observed during tumorigenesis, which is regulated by hypoxia-inducible factors (HIFs). HIFs maintain cancer stem cells stemness, and cancer stem cells could induce colon tumorigenesis [35]. EGFL6 has been reported that could be induced under hypoxia [36]. In this research, we found that hypoxia not only induce EGFL6 expression, but also ADAMTS1, an acute hypoxia-inducible gene [37]. From the results in this research, EGFL6 regulates STAT3, FAK and Src, which correlate to cancer stem cell proliferation (Fig. 4b, c) and self-renewal [22, 23]. Bai S’s study had also proven that EGFL6 promotes the asymmetric division of cancer stem-like cells in ovarian cancer [29], consistent with this research’s result that EGFL6 could keep the cell stemness by regulating the expression of cancer stem cell associated genes, POU5F1, NANOG, and LIN28 (Fig. 4d, e). These results provide evidences that EGFL6 expression correlate with CRC cancer stem cell.

One of the adverse events of bevacizumab is wound healing complications. Therefore, whether EGFL6-E5-IgG could contribute to better efficacy, but exert adverse effect becomes crucial. In the wound healing model, the wound closer rate is similar between EGFL6-E5-IgG-treated group and Control group. In contrast to these two groups, bevacizumab-treated group exhibited impaired healing, in which the wounds still moist and had inflammatory response, with high risk of infection due to long-term recovery. The results indicated that EGFL6-E5-IgG had no effect on wound healing also showed obvious tumor growth inhibitory capability in HCT-116 xenograft model. Similar results had also been obtained by Noh et al. [36]. In addition, EGFL6-E5-IgG exhibited significantly increase anti-metastasis ability, without significant difference compared to bevacizumab-treated group. Therefore, we conclude that EGFL6-E5-IgG has the ability to inhibit CRC growth, metastasis and has no visibly toxicity in *in vivo* model, which worth further investigation and development.

There are limitations in this study. The characteristic of CRC patients didn’t include the treatment type and comorbidities, so it’s difficult to validate the unknown factors that may potentially influence EGFL6 expression. In addition, whether EGFR or αvβ3 integrin contribute more to EGFL6 signaling still need further investigation. EGFL6-E5-IgG still need to optimize; current data showed its potential efficacy to treat colorectal cancer in mice. An animal model using EGFL6-E5-IgG compares to biologic targeted therapy drugs, such as bevacizumab plus chemotherapy, need to be conducted. Even though we did not see much side effects using EGFL6-E5-IgG, the safety issue regarding EGFL6-E5-IgG still need further experiments to validate.

## Conclusions

In summary, this research provided the evidence that hypoxia-induced EGFL6 activated EGFR/αvβ3 integrin signaling, brought FAK binding to Src. ERK, AKT, Cyclin D1 signaling were next activated to promotes cell proliferation. STAT3, ADAMTS1 and Snail were also induced to influence cell migration, invasion and cell self-renewal function in CRC. EGFL6 could be detected in the early stage of CRC patients and tumorigenesis mouse. Moreover, anti-EGFL6 antibody EGFL6-E5-IgG showed tumor growth inhibition and significantly influence anti-metastasis property *in vivo* without affecting wound healing, indicated EGFL6-E5-IgG is worthy of further development as a potential therapeutic agent against CRC. Taken together, this study demonstrated that EGFL6 could be a potential tumor target in CRC.

## Methods

### Cell culture and reagents

Human colorectal carcinoma cell lines HCT-116 and HT-29, and mouse colon carcinoma cell line CT-26 were purchased from Bioresource Collection and Research Center (BCRC, Hsinchu, Taiwan). HCT-116 and HT-29 cells were grown in McCoy’s 5A (Sigma-Aldrich, Darmstadt, Germany) and CT-26 was grown in RPMI-1640 (Gibco, Dublin, Ireland) supplemented with 4.5 g/L glucose, 10 mM HEPES, and 1.0 mM sodium pyruvate. All cells were maintained in humidified air containing 5% CO_2_ incubator at 37 °C and cultured every 2–3 days.

EGFL6 recombinant protein was purchased from Sino Biological (Beijing, China). EGFL6 therapeutic antibody (EGFL6-E5-IgG) was generated and provided by Dr. Yu-Ching Lee using phage display technology. Bevacizumab (Avastin®) was purchased from Genentech (California, USA). 5-FU (Fluorouracil® Injection) was purchased from Pfizer (New York City, USA).

### Patients and colon tissue specimens

Tissue biopsy samples used in Fig. 1a were collected after prior informed written consent as part of a study (no. N201701061) approved by the human ethics committee of Taipei Medical University Joint Institutional Review Board. No chemotherapy or radiation therapy was given to the enrolled patients before surgical therapy. The TNM stages were determined based on the American Joint Committee on Cancer/International Union Against Cancer TNM staging system. Tissue biopsy used in Fig. 1b is approved by Taipei Medical University Joint Biobank (TMU-JBB) (no. N201703080).

### In vivo animal models

All experiments on mice were performed in accordance with institutional and national guidelines and regulations. Protocols have been reviewed and approved by Animal Use and Management Committee of Taipei Medical University (IACUC approved No. TMU LAC-2016-0034). Mice were maintained under a 12:12 h light: dark cycle and fed with standard diet and water ad libitum.

For Azoxymethane (AOM)—induced mutagenesis assay, a total of 35 eight-week-old A/J mice (Jackson Laboratories, Bar Harvor, ME) were randomly divided into Control or AOM treatment group. AOM (A5486-25MG, Sigma-Aldrich, Darmstadt, Germany) was freshly diluted to 10 mg/kg, then add 1125 mL sterile isotonic saline to the concentration of 1 mg/mL. Mice were injected with 10 mg/kg AOM intraperitoneally (i.p.) once a week for 6 consecutive weeks as previously described [38, 39]. Mice were sacrificed and colons were collected at 1, 2, 4, 8, and 18 weeks after the last AOM challenge, a section of the column (from anus to the cecum) was taken out from mice, after wash, reserved in 10% formalin in 4 °C. After one day, methylene blue, H&E staining, and immunohistochemistry (IHC) staining were used to assess EGFL6.

For anti-cancer activity xenograft model, a total of 24 six-week-old nude mice (National Laboratory Animal Center (NLAC), Taipei, Taiwan) were injected subcutaneously with the same volume of Matrigel (BD bioscience, San Jose, California, USA), and 1$\times$10^7^ of HCT-116 cells into the right flank of each animal. When tumors had grown to around 300 mm^3^, the treatment started. Tumor size was measured twice weekly and calculated from V = length$\times$width^2^/2. Tumor growth inhibition (TGI%) = [1 − (T_t_ − T_0_)/(C_t_ − C_0_)$\times$100], where C_0_ and C_t_ are mean tumor volumes of the Control group by first data point and day t, respectively; while T_0_ and T_t_ are mean tumor volumes of treatment group by first data point and day t.

For lung metastasis animal model, 8-week-old balb/c mice (NLAC, Taipei, Taiwan) were injected with CT-26 cells (2$\times$10^5^ cell/mouse) intravenously and then divided into three groups for treatment. After 3 weeks of treatment, animals were sacrificed and lung nodules were counted for data analysis.

For wound healing animal model, after given narcotic and sterilized with iodine, 4.0-mm circular full-thickness skin excision wound were created on the dorsal site of each nude mice using biopsy punch. A Tegaderm film was attached to the wound to protect and avoid scab generation. The mice were then randomly divided into three groups for treatments. The wound area and body weight were measured every other day until wound closure.

For angiogenesis animal model, mice were injected with 500 µl matrigel subcutaneously mixed with endothelial growth factor (EGF, 150 ng/mL) and heparin (10 µl), treated anti-EGFL6 antibody (15 mg/kg, q5D) through intravenous injection immediately after subcutaneous injection. After seven-day treatment, animals were sacrificed and the matrigel were carefully dissected. Hemoglobin content was then analyzed by Drabkin’s reagent kit (Sigma Chemical, St. Louis, MO, USA) to quantify the blood vessel formation.

### RNA isolation and quantitative real‐time PCR

The RNA was isolated using the TRIzol reagent and the Direct-zol RNA MiniPrep (ZYMO research, Irvine, CA, USA) according to the manufacturer’s instruction. Reverse transcription reactions were performed using the RT Kit (Takara, USA) with 2 µg of total RNA according to the manufacturer’s instruction. Quantitative real-time PCR was performed by ABI StepOnePlus Real-Time PCR Systems using SYBR Green dye (Life Technologies, Grand Island, NY, USA). Relative RNA abundance was calculated using the ^ΔΔ^C_T_ formula and normalized to the transcript levels of the housekeeping gene GAPDH. Primer sequences used for quantitative real-time PCR were listed in Additional file 2: Table S1.

### Western blotting

Total protein was extracted by homogenization in ice-cold RIPA buffer containing protease and phosphatase inhibitor cocktail. Equal amounts of protein extracts were heated in sample buffer and then separated by SDS-polyacrylamide gel electrophoresis. Separated proteins were then transferred to PVDF membrane. The membranes were subsequently probed using the following primary antibodies listed in Additional file 2: Table S2–S3. Immunoreactive bands were visualized with an enhanced chemiluminescence substrate detection kit (Amersham, Buckinghamshire, UK).

### Colony formation assay

For determination of colony forming units (CFUs), cells were plated at a density of 300 cells/9.01 cm^2^ culture dish. After 8 days of incubation, the colonies formed were fixed with ice-cold methanol for 10 min, and then stained with Giemsa solution for 15 min. After washing and drying, the colony numbers were calculated.

### siRNA transfection

Cells were transfected with the siRNA targeting *EGFL6*, *HIF-1α* and scrambled siRNA (siEGFL6 #10: #1,299,003, HIF-1α: #42,840, scrambled siRNA: #4,390,847, Thermo Fisher Scientific) using RNAiMAX Transfection Reagent according to the manufacturer’s protocol. The comparison of different siEGFL6 in HCT116 and HT29 were analyzed in Additional file 1: Figure S6.

### Migration and invasion assays

Cell migration and invasion abilities were evaluated in transwell with 8-µm pore size polycarbonate membranes in 24-well plates (Corning Inc., Corning, NY, USA). The 1$\times$10^5^ cells were seeded to each transwell insert, and filled each well with culture medium. For invasion assays, the membranes in transwell were pre-coated with 50 µg of Matrigel to form matrix barriers. After incubation for 16 h, the cells remaining on the upper surfaces of the membrane were cleaned. The cells moving to the lower surfaces of the membrane were fixed with ice-cold 10% formalin for 10 min, stained with 0.2% crystal violet for 15 min and counted under a light microscope.

### Construction of chicken scFv library and biopanning

Chicken scFv library was constructed according to the published protocol with minor modifications [40]. Detail methods are described in Additional file 3: Supplementary materials.

### Statistical analysis

Each experiment was performed independently at least three times and the data were presented as mean $\pm$ standard error. Student’s *t*-test was used to analyze the data between the two groups. Non-parameter ANOVA and multiple comparisons test were used to analyze data between more than 3 groups. Fisher’s exact test was used to analyze the relationship between EGFL6 expression and CRC. A p-value of less than 0.05 was defined as a statistically significant difference.

## Supplementary Information

**Additional file 1: Figure S1.** EGFL6 expresses in CRC cells instead of normal colon epithelial cell. (A) The protein expression of EGFL6 (Epidermal growth factor-like protein 6) in normal colon epithelial cell and CRC (colorectal cancer) cells. The arrow indicates the EGFL6 protein’s location. (B) The EGFL6 mRNA expression in CRC cell lines. (C) The quantification of EGFL6 and EGFR (epidermal growth factor receptor) expression of all colon cell lines. All proteins were normalized with GAPDH.** Figure S2.** The quantification of protein expression. (A, B) Western blot quantified from Fig. 2E, H. (C, D) Western blot quantified from Fig. 4B, C. All phospho-proteins were normalized with their total-protein then were normalized with GAPDH.** Figure S3.** Protein expression reduction rate from siEGFL6 treatment in Figure 2H. All phospho-proteins were normalized with their total-protein then were normalized with GAPDH.** Figure S4.** The relative mRNA level of HT29 treated by siEGFL6. Analysis MMP-2 (matrix metalloproteinase-2), ADAMTS1 (a disintegrin and metalloprotease with thrombospondin motif 1) and Snail expression by treated siEGFL6 #10, data generated from qPCR and normalized with GAPDH. Two-way ANOVA with Sidak’s multiple comparisons test was used in statistical analysis. * p < 0.05.** Figure S5.** The binding affinity of EGFL6-E5-IgG. Binding curves (black thin line) and the sensor gram traces (blue, red and black thick line) exemplifying association / dissociation kinetics of scFv E5 to the immobilized EGFL6 recombinant protein as the graph shown. The scFv E5 concentrations are 50 µg/mL (red), 100 µg/mL (black), and 400 µg/mL (blue). Data was fit with 1:1 binding interaction model with errors from TraceDrawer.** Figure S6.** The function of anti-EGFL6 antibodies in HCT116. (A) Cell proliferation inhibition test treated by EGFL6-E5-IgG (50 μg/mL). The assay was performed by MTT, 3000 cell number was seeded into 96 well. (B) Colony formation test treated by EGFL6-E5-IgG (25 μg/mL), 300 cell number was seeded into 6 well for CFU assay. (C) Cell migration inhibition test treated by EGFL6-E5-IgG. ** p < 0.01.** Figure S7.** Anticancer activity of EGFL6 antibody in human glioblastoma U87 xenograft model. A total of 18, twelve-week-old nude mice were injected intravenously with the same volume of Matrigel, and 1×107 of U87 cells into the right flank of each animal. The tumor volume and body weight observation in U87 xenograft model treated with three groups: control (IgG, iv, qwk, n=6), EGFL6-E5-IgG (10 mg/kg, iv, qwk, n=6) and EGFL6-E5-IgG (20 mg/kg, iv, qwk, n=6).

**Additional file 2: Table S1.** Primers sequences for quantitative real-time PCR.** Table S2.** Primary antibodies for western blot.** Table S3.** Secondary antibodies for western blot.

**Additional file 3.** Supplementary Materials and Methods: surface plasmon resonance; Construction of chicken scFv library and biopanning; Methylene blue staining; 3-(4, 5-dimethylithiazol-2-yl)-2, 5-diphenyl tetrazolium bromide (MTT) assay.

## Data Availability

The datasets used and analyzed during the current study are available from the corresponding author on reasonable request.

## Abbreviations

5-FU: 5-Fluoracil
ADAMTS1: A disintegrin and metalloprotease with thrombospondin motif 1
AKT: Protein kinase B
AOM: Azoxymethane
BSA: Bovine serum albumin
CB-EGF: Ca2 + binding EGF
CFUs: Colony forming units
CRC: Colorectal cancer
mCRC: Metastasis colorectal cancer
DMSO: Dimethyl sulfoxide
ECM: Extracellular matrix
EGFL6: Epidermal growth factor-like protein 6
EGFR: Epidermal growth factor receptor
ELISA: Enzyme-linked immunosorbent assay
ERK: Extracellular signal–regulated kinase
FAK: Focal adhesion kinase
GAPDH: Glyceraldehyde-3-Phosphate Dehydrogenase
H&E stain: Hematoxylin and eosin stain
HIF-1α: Hypoxia-inducible factor-1 alpha
HRP: Horseradish peroxidase
IHC: Immunohistochemistry
KD: Dissociation constant
MTT: 3-(4,5-dimethylthiazol-2-yl)-2,5-diphenyltetrazolium bromide
MMP-2: Matrix metalloproteinase-2
MMP-9: Matrix metalloproteinase-9
NBF: Neutral buffered formalin
PBS: Phosphate-buffered saline
PCR: Polymerase chain reaction
POU5F1: : POU class 5 homeobox 1
PVDF: Polyvinylidene fluoride
RGD: Arginine-glycine-aspartic acid
RIPA: Radioimmunoprecipitation assay
RTKs: Receptor tyrosine kinases
scFv: Single-chain variable fragment
SDS: Sodium dodecyl sulfate
SEM: Standard error of the mean
STAT3: Signal transducer and activator of transcription 3
TGI: Tumor growth inhibition
VEGF: Vascular endothelial growth factor
