# Supplementary material for: EGFL6 promotes colorectal cancer cell growth and mobility and the anti‐cancer property of anti-EGFL6 antibody
Source: Cell Biosci. 2021 Mar 16;11:53. doi: 10.1186/s13578-021-00561-0 (PMC7962215; doi:10.1186/s13578-021-00561-0)
Supplement: Supplementary file 1 — Additional file 1: Figure S1. EGFL6 expresses in CRC cells instead of normal colon epithelial cell. (A) The protein expression of EGFL6 (Epidermal growth factor-like protein 6) in normal colon epithelial cell and CRC (colorectal cancer) cells. The arrow indicates the EGFL6 protein’s location. (B) The EGFL6 mRNA expression in CRC cell lines. (C) The quantification of EGFL6 and EGFR (epidermal growth factor receptor) expression of all colon cell lines. All proteins were normalized with GAPDH. Figure S2. The quantification of protein expression. (A, B) Western blot quantified from Fig. 2E, H. (C, D) Western blot quantified from Fig. 4B, C. All phospho-proteins were normalized with their total-protein then were normalized with GAPDH. Figure S3. Protein expression reduction rate from siEGFL6 treatment in Figure 2H. All phospho-proteins were normalized with their total-protein then were normalized with GAPDH. Figure S4. The relative mRNA level of HT29 treated by siEGFL6. Analysis MMP-2 (matrix metalloproteinase-2), ADAMTS1 (a disintegrin and metalloprotease with thrombospondin motif 1) and Snail expression by treated siEGFL6 #10, data generated from qPCR and normalized with GAPDH. Two-way ANOVA with Sidak’s multiple comparisons test was used in statistical analysis. * p < 0.05. Figure S5. The binding affinity of EGFL6-E5-IgG. Binding curves (black thin line) and the sensor gram traces (blue, red and black thick line) exemplifying association / dissociation kinetics of scFv E5 to the immobilized EGFL6 recombinant protein as the graph shown. The scFv E5 concentrations are 50 µg/mL (red), 100 µg/mL (black), and 400 µg/mL (blue). Data was fit with 1:1 binding interaction model with errors from TraceDrawer. Figure S6. The function of anti-EGFL6 antibodies in HCT116. (A) Cell proliferation inhibition test treated by EGFL6-E5-IgG (50 μg/mL). The assay was performed by MTT, 3000 cell number was seeded into 96 well. (B) Colony formation test treated by EGFL6-E5-IgG (25 μg/mL), 300 [file 13578_2021_561_MOESM1_ESM.docx]

**Additional file 1. Supplementary Figures**

**
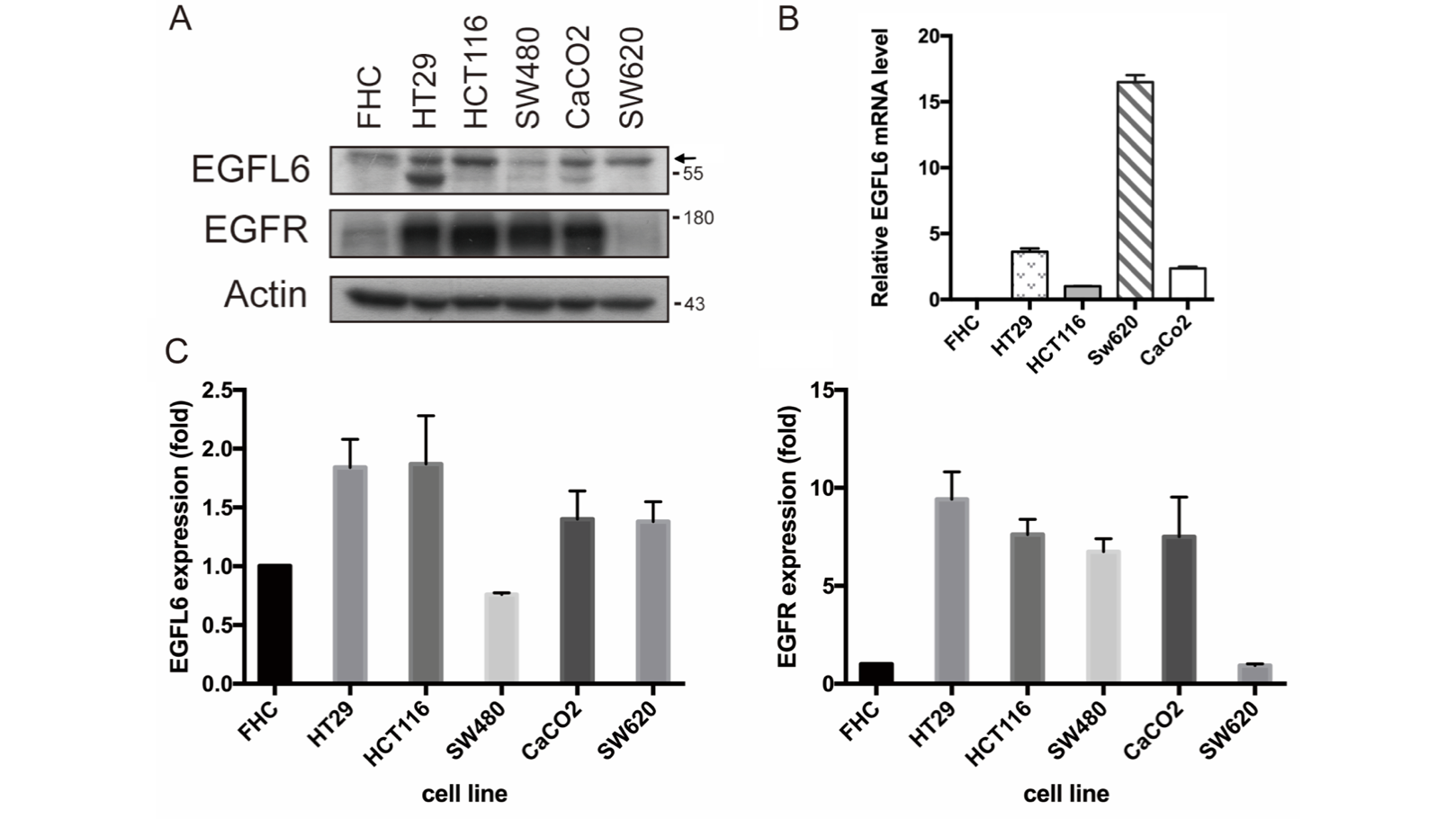
**

**Figure S1. EGFL6 expresses in CRC cells instead of normal colon epithelial cell.** (A) The protein expression of EGFL6 (Epidermal growth factor-like protein 6) in normal colon epithelial cell and CRC (colorectal cancer) cells. The arrow indicates the EGFL6 protein’s location. (B) The EGFL6 mRNA expression in CRC cell lines. (C) The quantification of EGFL6 and EGFR (epidermal growth factor receptor) expression of all colon cell lines. All proteins were normalized with GAPDH.


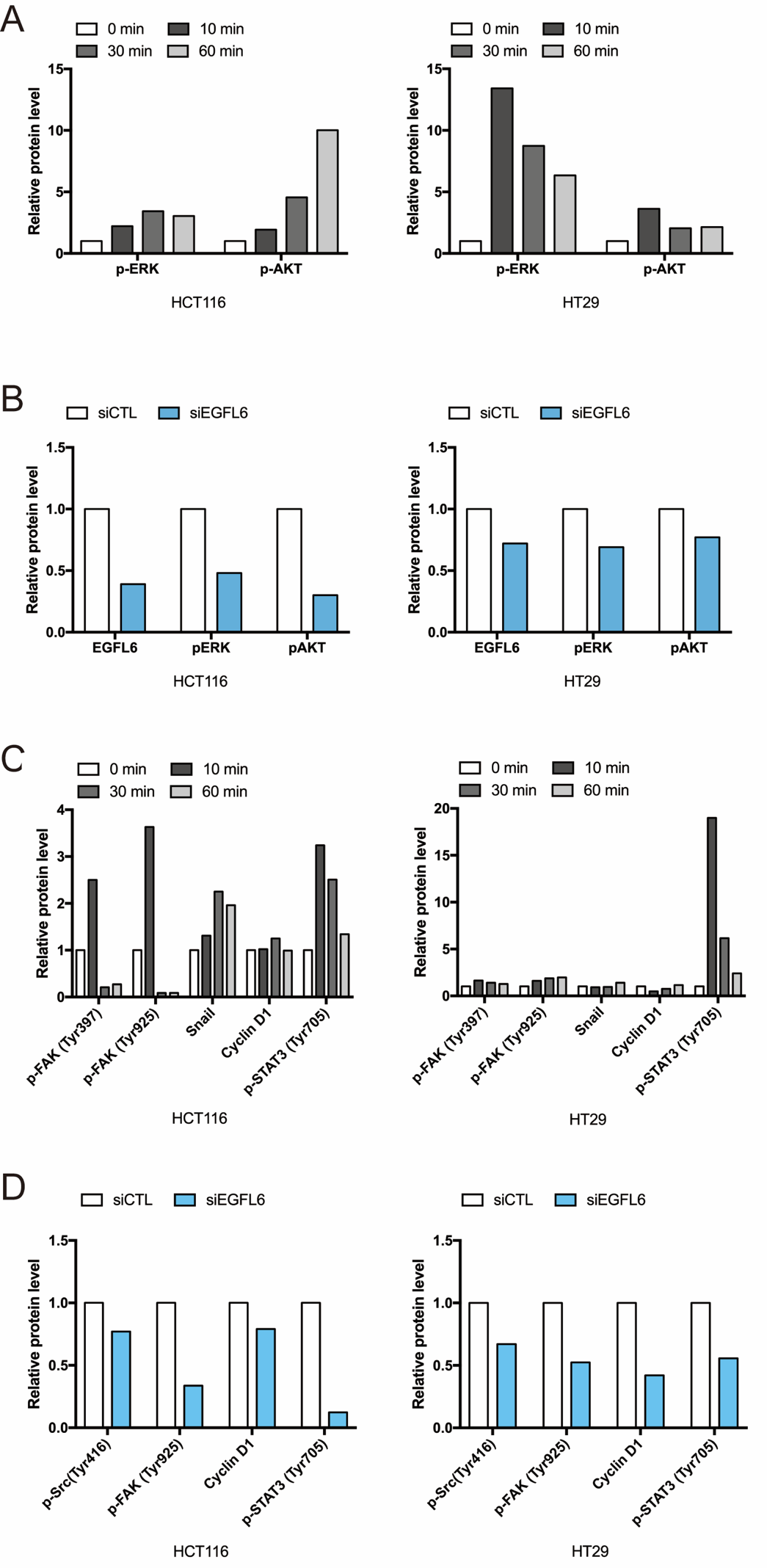


**Figure S2. The quantification of protein expression.** (A, B) Western blot quantified from Fig. 2E, H. (C, D) Western blot quantified from Fig. 4B, C. All phospho-proteins were normalized with their total-protein then were normalized with GAPDH.

**
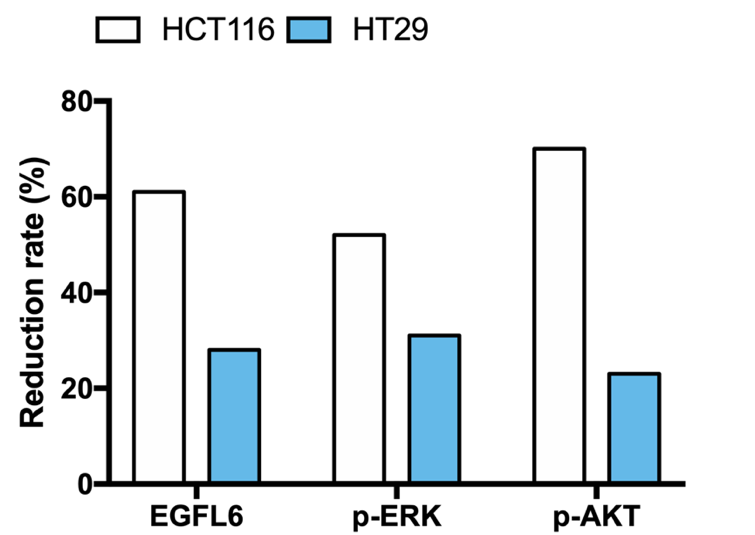
**

**Figure S3. Protein expression reduction rate from siEGFL6 treatment in Figure 2H.** All phospho-proteins were normalized with their total-protein then were normalized with GAPDH.


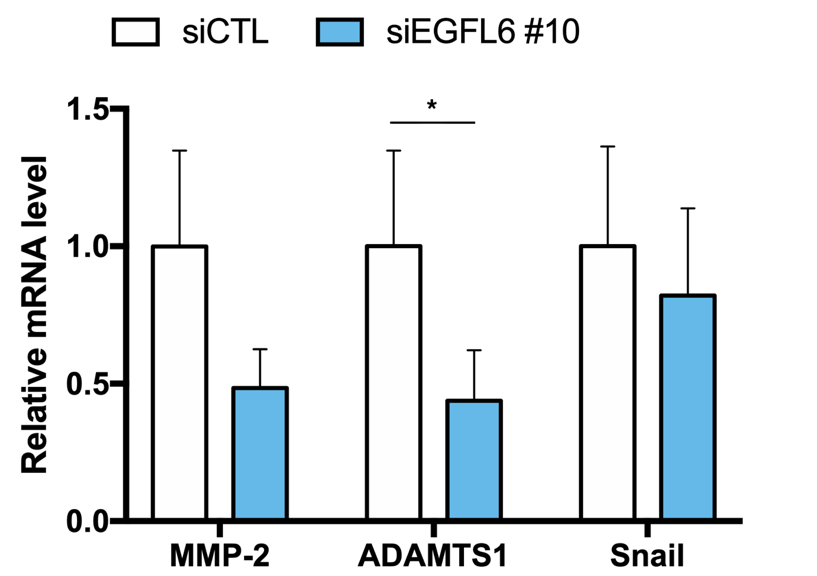


**Figure S4. The relative mRNA level of HT29 treated by siEGFL6.** Analysis *MMP-2 (*matrix metalloproteinase*-2), ADAMTS1 (a disintegrin and metalloprotease with thrombospondin motif 1)* and *Snail* expression by treated siEGFL6 #10, data generated from qPCR and normalized with GAPDH. Two-way ANOVA with Sidak’s multiple comparisons test was used in statistical analysis. * *p* < 0.05.

**
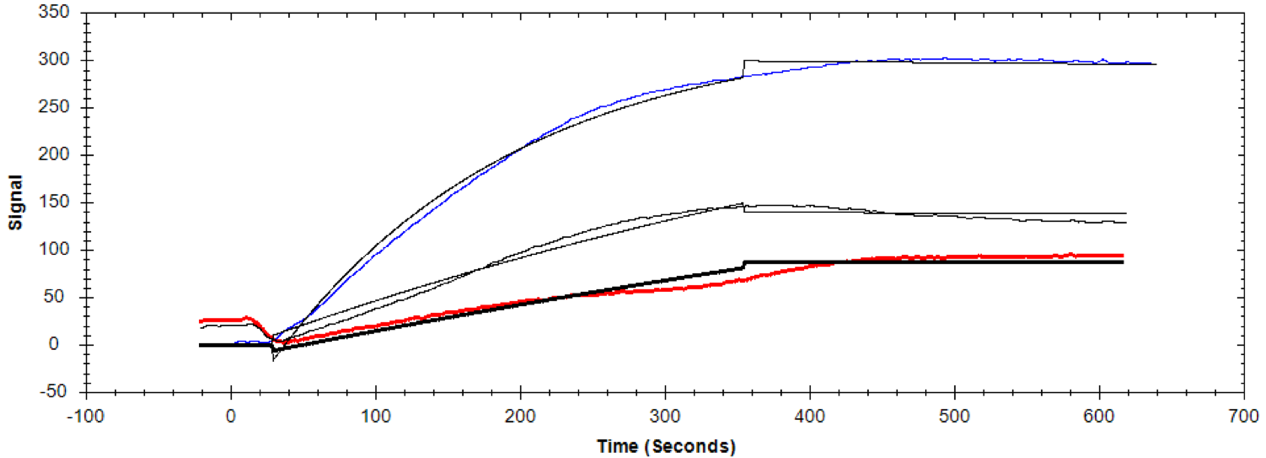
**

**Figure S5. The binding affinity of EGFL6-E5-IgG.** Binding curves (black thin line) and the sensor gram traces (blue, red and black thick line) exemplifying association / dissociation kinetics of scFv E5 to the immobilized EGFL6 recombinant protein as the graph shown. The scFv E5 concentrations are 50 µg/mL (red), 100 µg/mL (black), and 400 µg/mL (blue). Data was fit with 1:1 binding interaction model with errors from TraceDrawer.


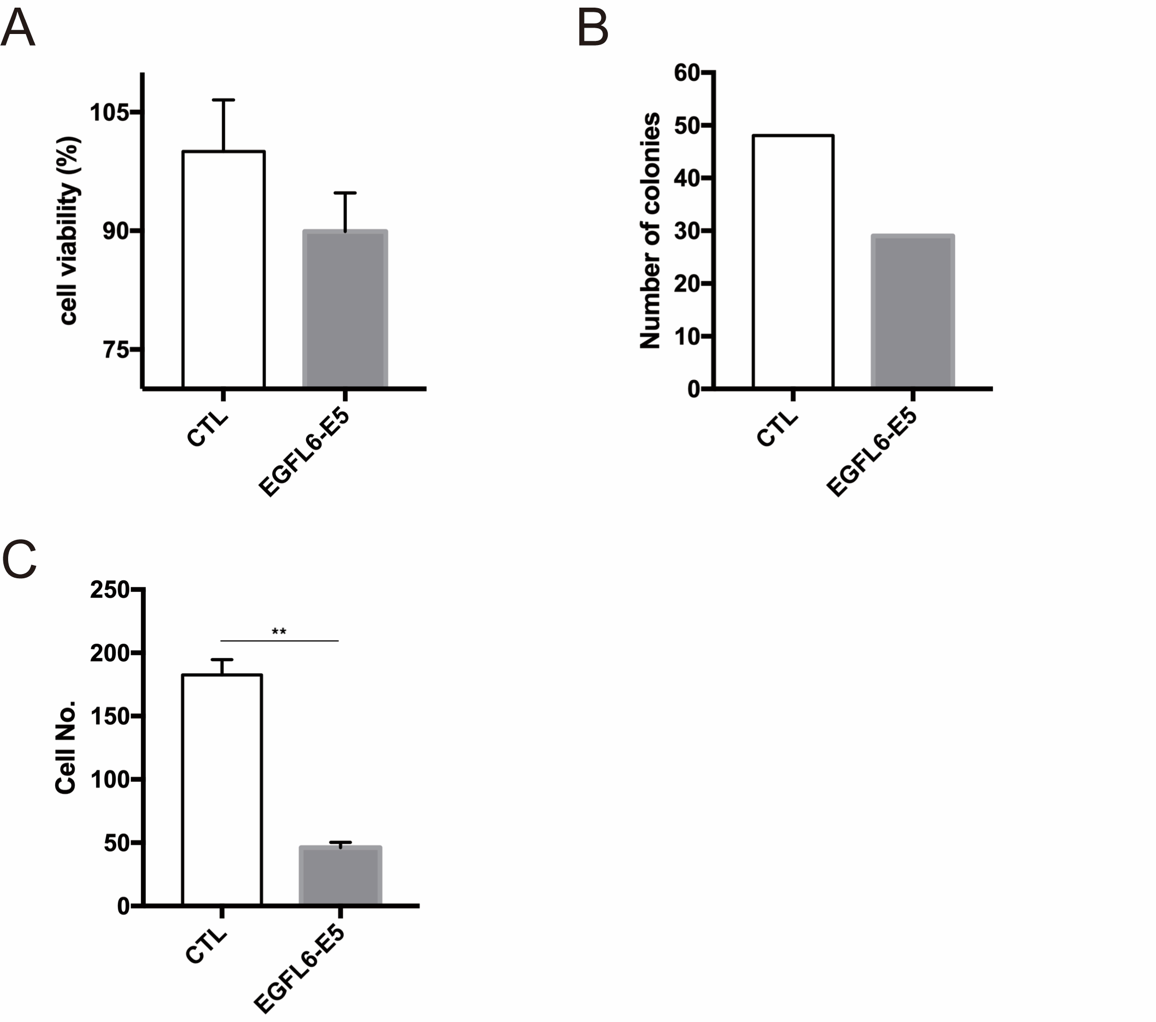


**Figure S6. The function of anti-EGFL6 antibodies in HCT116.** (A) Cell proliferation inhibition test treated by EGFL6-E5-IgG (50 $\mu$g/mL). The assay was performed by MTT, 3000 cell number was seeded into 96 well. (B) Colony formation test treated by EGFL6-E5-IgG (25 $\mu$g/mL), 300 cell number was seeded into 6 well for CFU assay. (C) Cell migration inhibition test treated by EGFL6-E5-IgG. ** *p* < 0.01.

**
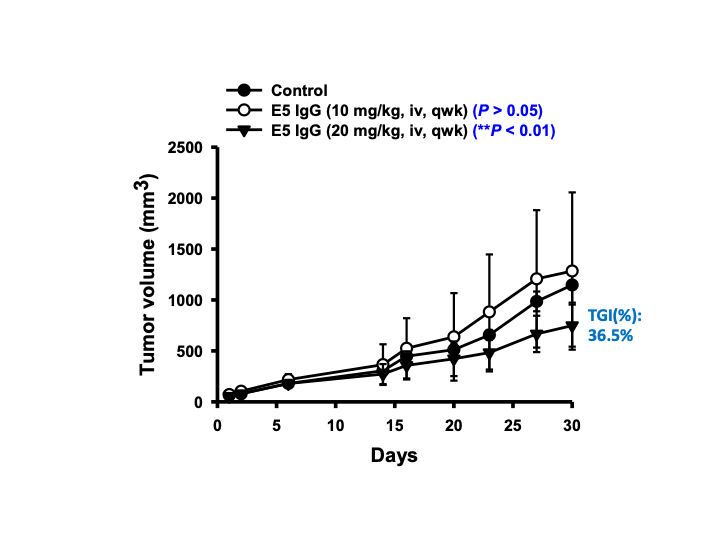
**

**Figure S7. Anticancer activity of EGFL6 antibody in human glioblastoma U87 xenograft model.** Ａ total of 18, twelve-week-old nude mice were injected intravenously with the same volume of Matrigel, and 1$\times$10^7^ of U87 cells into the right flank of each animal. The tumor volume and body weight observation in U87 xenograft model treated with three groups: control (IgG, iv, qwk, n=6), EGFL6-E5-IgG (10 mg/kg, iv, qwk, n=6) and EGFL6-E5-IgG (20 mg/kg, iv, qwk, n=6).
