# Supplementary material for: EGFL6 promotes colorectal cancer cell growth and mobility and the anti‐cancer property of anti-EGFL6 antibody
Source: Cell Biosci. 2021 Mar 16;11:53. doi: 10.1186/s13578-021-00561-0 (PMC7962215; doi:10.1186/s13578-021-00561-0)
Supplement: Supplementary file 2 — Additional file 2: Table S1. Primers sequences for quantitative real-time PCR. Table S2. Primary antibodies for western blot. Table S3. Secondary antibodies for western blot. [file 13578_2021_561_MOESM2_ESM.docx]

**Additional file 2: Supplementary Tables**

**Table S1. Primers sequences for quantitative real-time PCR**

| Target genes | Primer sequences |
| --- | --- |
| *GAPDH* (Forward) | 5’-GATTCCACCCATGGCAAATTC-3’ |
| *GAPDH* (Reverse) | 5’-CTGGAAGATGGTGATGGGATT-3’ |
| *EGFL6* (Forward) | 5’-TTGGCAGGTCACAAGAAAGA-3’ |
| *EGFL6* (Reverse) | 5’-GCCGGTAATCAAAGAGCAAAC-3’ |
| *POU5F1* (Forward) | 5’-GGAGGAAGCTGACAACAATGA-3’ |
| *POU5F1* (Reverse) | 5’-CTCTCACTCGGTTCTCGATACT-3’ |
| *NANOG* (Forward) | 5’-TCCTGAACCTCAGCTACAAAC-3’ |
| *NANOG* (Reverse) | 5’-GCGTCACACCATTGCTATTC-3’ |
| *LIN28* (Forward) | 5’-CAGAGTGGAGAAAGTGGGAATAG-3’ |
| *LIN28* (Reverse) | 5’-CTAGAGGGAAGAAAGGGTGATG-3’ |
| *MMP-2* (Forward) | 5’-ACT GTG ACG CCA CGT GAA CAA-3’ |
| *MMP-2* (Reverse) | 5’-CGT ATA CCG CAT CAA TCT TTT CC-3’ |
| *MMP-9* (Forward) | 5’-GCA CGA CGT CTT CCA GTA CC-3’ |
| *MMP-9* (Reverse) | 5’-CAG GAT GTC ATA GGT CAC GTA GC-3’ |
| *ADAMTS1* (Forward) | 5’-ACG AGT GCG CTA CAG ATC CT-3’ |
| *ADAMTS1* (Reverse) | 5’-CAG CGT ACT TGG GAA TCC AT-3’ |
| *Snail* (Forward) | 5’-TAC TGC AAC AAG GAA TAC CTC-3’ |
| *Snail* (Reverse) | 5’-GTA CTT CTT GAC ATC TGA GTG G-3’ |
| *HIF-1α* (Forward) | 5’-ATC CAT GTG ACC ATG AGG AAA TG-3’ |
| *HIF-1α* (Reverse) | 5’-TCG GCT AGT TAG GGT ACA CTT C-3’ |

**Table S2. Primary antibodies for western blot**

| Antibodies | Ratio | Brand |
| --- | --- | --- |
| EGFL6 | 1:1000 | ab167281, Abcam |
| GAPDH | 1:10000 | MAB374, Millipore |
| p-ERK | 1:2000 | cs-9101, Cell Signaling Technology |
| ERK | 1:3000 | cs-9102, Cell Signaling Technology |
| p-AKT | 1:1000 | cs-4060, Cell Signaling Technology |
| AKT | 1:1000 | cs-9272, Cell Signaling Technology |
| HIF-1a | 1:1000 | 610969, BD Biosciences |
| p-FAK (Y397) | 1:500 | cs-3284, Cell Signaling Technology |
| p-FAK (Y925) | 1:500 | cs-3284, Cell Signaling Technology |
| FAK | 1:1000 | cs-3285, Cell Signaling Technology |
| Snail | 1:1000 | cs-3879, Cell Signaling Technology |
| Cyclin D1 | 1:1000 | CC12, EMD Millipore |
| p-STAT3 | 1:2000 | cs-9145, Cell Signaling Technology |
| STAT3 | 1:5000 | BD610190, BD Biosciences |
| p-Src | 1:1000 | cs-21012, Cell Signaling Technology |

**Table S3. Secondary antibodies for western blot**

| Antibody | Ratio | Brand |
| --- | --- | --- |
| goat anti-mouse horseradish peroxidase (HRP) conjugate | 1:5000 | sc-2005, Santa Cruz Biotechnology |
| goat anti-rabbit HRP conjugate | 1:5000 | sc-2004, Santa Cruz Biotechnology |
