# Supplementary material for: EGFL6 promotes colorectal cancer cell growth and mobility and the anti‐cancer property of anti-EGFL6 antibody
Source: Cell Biosci. 2021 Mar 16;11:53. doi: 10.1186/s13578-021-00561-0 (PMC7962215; doi:10.1186/s13578-021-00561-0)
Supplement: Supplementary file 3 — Additional file 3. Supplementary Materials and Methods: surface plasmon resonance; Construction of chicken scFv library and biopanning; Methylene blue staining; 3-(4, 5-dimethylithiazol-2-yl)-2, 5-diphenyl tetrazolium bromide (MTT) assay. [file 13578_2021_561_MOESM3_ESM.docx]

**Supplementary Materials and Methods**

*Surface plasmon resonance*

The binding affinity of E5-IgG to EGFL6 was analyzed by an OpenSPR instrument (Nicoya Lifesciences, Kitchener ON, Canada). Recombinant EGFL6 molecule served as the ligand and was immobilized on a Ni-NTA sensor chip. The prepared ligand solution was introduced into a sensor chip with PBS, as the running buffer. After ligand coating, the analyte E5-IgG was tested in different concentrations in PBS, as the running buffer for the reaction. Following the analysis of each E5-IgG concentration, 10 mM Glycine-HCl, PH2.2 was injected to regenerate the chip. Analysis of the data was made using Trace Drawer software according to a 1:1 binding model.

*Construction of chicken scFv library and biopanning*

A phage library displaying the scFv antibody was constructed according to the published protocol with minor modifications. Briefly, chicken spleens were harvested 7 days after the final immunization. After homogenization, total RNA was extracted and reverse transcribed into the first-strand cDNA by using a SuperScript RT kit (Invitrogen, USA). After amplification using chicken-specific primers, gene products of heavy-chain and light-chain variable (VH and VL, respectively) regions were subjected to a second round of polymerase chain reaction (PCR) with a linker to form full-length scFv molecules, which were cloned further into the pComb3X vector. The recombinant DNA was transformed into the Escherichia coli ER2738 strain through electroporation, and the VCS-M13 (Stratagene, USA) helper phage was then added to initiate recombinant phage production. The total phage library was precipitated with 4% polyethylglycol 8000 and 3% NaCl (w/v) and resuspended in PBS containing 1% bovine serum albumin (BSA) and stored at 4°C.

For biopanning, 10^11^ recombinant phage particles from the constructed scFv antibody library were added to a microtiter plate precoated with a recombinant EGFL6 protein (1 µg/well) and incubated at room temperature for 2 h. Next, unbound phage particles were removed and the well was washed with PBST (PBS with 0.05% Tween 20) for 10 times. Bound phage particles were eluted with 0.1 M HCl/glycine (pH 2.2)/0.1% BSA elution buffer, neutralized with 2 M Tris base buffer, and immediately used to infect the Escherichia coli ER2738 strain for recombinant phage amplification. Phage particles were precipitated and recovered as described previously and used for the next round of panning. The panning procedure was repeated four times. After panning, enriched scFv clones were selected for single-colony analysis. These scFv clones were expressed in Escherichia coli TOP 10F’ (Invitrogen, a nonsuppressor strain) and purified with Ni^2+^-charged sepharose according to the manufacturer’s instructions (GE Biosciences, UK).

*Methylene blue staining*

Colons were collected at the indicated time, longitudinally opened and fixed in 10% neutral buffered formalin (NBF) at 4^o^C overnight. Fixed tissues were rinsed with phosphate-buffered saline (PBS), stained with 0.05% methylene blue, washed with PBS for 5 min and then photographed.

*3-(4, 5-dimethylithiazol-2-yl)-2, 5-diphenyl tetrazolium bromide (MTT) assay*

The cell proliferation ability was evaluated using MTT. Cells were incubated with culture medium containing 0.5 mg/mL MTT for 1 h at 37 °C under humidified 5% CO_2_ in air. After incubation, cells were lysed by dimethyl sulfoxide (DMSO) and then the absorbance was measured by an enzyme-linked immunosorbent assay (ELISA) reader at 550 nm wavelength (Synergy™ HTX, BioTek).
